# Clinical Features, CT Imaging Decisions and Yield by Age in Adults With Abdominal Pain in the Emergency Department

**DOI:** 10.1111/acem.70299

**Published:** 2026-05-10

**Authors:** Ari B. Friedman, Michael N. Adjei‐Poku, Liliana A. Schadt, Yang Li, Anne R. Cappola, Rachel R. Kelz, Ula Hwang, Gopal Kowdley, Aubrey Mwinyogle, N. Seth Trueger

**Affiliations:** ^1^ Department of Emergency Medicine University of Pennsylvania Philadelphia Pennsylvania USA; ^2^ Leonard Davis Institute of Health Economics University of Pennsylvania Philadelphia Pennsylvania USA; ^3^ Department of Medical Ethics and Health Policy University of Pennsylvania Philadelphia Pennsylvania USA; ^4^ School of Nursing University of Pennsylvania Philadelphia Pennsylvania USA; ^5^ Division of Endocrinology, Diabetes, and Metabolism Hospital of the University of Pennsylvania Philadelphia Pennsylvania USA; ^6^ Department of Surgery University of Pennsylvania Philadelphia Pennsylvania USA; ^7^ Department of Emergency Medicine NYU Grossman School of Medicine New York City New York USA; ^8^ James J Peters VA Medical Center New York USA; ^9^ NYU Langone Health New York USA; ^10^ Tidal Health General Surgery, Tidal Health Salisbury Maryland USA; ^11^ Vascular Surgery, Faith Regional Health Services Norfolk Nebraska USA; ^12^ Department of Emergency Medicine Feinberg School of Medicine Northwestern University Chicago Illinois USA; ^13^ JAMA Network Open Chicago Illinois USA

## Abstract

**Objectives:**

Older adults with abdominal pain present diagnostic uncertainty due to less informative histories/exams, broader etiologies, and higher morbidity. Whether ED imaging decisions are calibrated to this risk is unclear. The objective of this study was to compare age‐stratified clinical features, CT utilization, and CT diagnostic yield, and to assess how history/physical and clinician pretest suspicion relate to adverse outcomes.

**Methods:**

This was a retrospective cohort analysis of data from a prospective cohort collected from March 2016–January 2017 at a single community teaching hospital emergency department in southwest Baltimore. We analyzed 1169 visits of adults presenting with nontraumatic abdominal pain including 229 (19.6%) aged ≥ 60 years. Patients < 18 years were excluded. Age groups were 18–39, 40–59, ≥ 60 years. Outcomes were CT ordering, acute actionable CT findings, admission, surgery, and a composite of adverse outcomes (any actionable CT finding, admission, surgery, or Emergency General Surgical diagnosis). History and physical examination operating characteristics (e.g., sensitivity/specificity of tenderness, rebound) were also calculated.

**Results:**

Of 1169 visits, 19.6% were aged ≥ 60 years. CT ordering increased with age (41.7%, 66.2%, 70.7% for 18–39, 40–59, ≥ 60; *p* < 0.001), as did CT yield (18.4%, 31.2%, 37.7%; *p* < 0.001). Admissions (12.1%, 28.0%, 37.6%) and surgeries (4.6%, 9.0%, 10.6%) also rose with age. Clinician pretest suspicion was similar across age groups. Abdominal tenderness was less sensitive for adverse outcomes in older adults (sensitivity 0.58 in ≥ 60 vs. 0.73 in 18–39 and 0.73 in 40–59), while rebound tenderness was highly specific across ages (specificity 0.98, 0.96, 0.98). The number of potential diagnoses to consider rose with age.

**Conclusion:**

In this cohort, CT use and positivity increased with age and key exam findings (e.g., tenderness) being less informative in older adults, despite similar reported clinician pretest suspicion. These results support age‐aware imaging decisions and motivate reframing ED abdominal pain as a geriatric‐specific chief complaint.

## 1 Introduction

Abdominal pain is the most common chief complaint among patients in the emergency department (ED), with an estimated 12.4 million visits in 2021 [1]. From 2007 to 2019, ED visits for abdominal pain increased among every age group, with both CT imaging and CT yield (the proportion of tests which are positive, “test positivity”) increasing over time both across and within age groups [2]. This is compounded by the increase in ED visits by patients 65 years and older, which grew from 15% in 2011 to 25% in 2021 [1]. Historically, older patients with abdominal pain in the ED have high rates of hospital admission, surgical intervention, return visits, and mortality [2, 3, 4, 5, 6].

Across all conditions, it has been described that older adults, including older adults with abdominal pain, present with less specific symptoms due to physiologic changes, such as age‐related changes in nociception [7], which may make diagnoses more difficult, and that older patients may present later with more advanced disease [8, 9, 10]. Older adults may also show fewer physical signs of illness. They may be less likely to mount a fever or have abdominal tenderness on physical exam [11, 12, 13]. Additionally, older adults are hypothesized to present with a broader range of underlying etiologies for each presentation as they have accumulated more deficits to multiple body systems on average [8, 14, 15]. For instance, accumulated atherosclerosis drives presentations of mesenteric ischemia—which is rarely seen in younger adults [16]. These geriatric hypotheses are underpinned by copious clinical experience, with some supportive data, but have historically been difficult to test empirically. Not only is there more uncertainty when diagnosing older adults with acute illness, but there is also ambiguity about the degree(s) of uncertainty.

Previous research across all adults and disease processes places considerable value on the role of the history of present illness in making a diagnosis, with 73% of final general medical clinic diagnoses being accurately made with history and physical exam alone [17]. The precise utility of historical elements and physical examination findings, as well as vital signs, clinical gestalt, and diagnostic testing, remains unclear.

To bring evidence to these geriatric hypotheses, we utilize abdominal pain as a model system. Historically, a chief complaint of abdominal pain in the emergency department (ED) conveys subsequent morbidity and mortality of 5%–10% or more [18, 19], comparable to a ST‐elevation myocardial infarction [20], with higher morbidity among those patients who survive [19]. By contrast, in younger adults presenting to the ED for abdominal pain, morbidity/mortality is less severe [7, 8, 14, 21]. Abdominal pain presentations to the ED have simple testing available in almost all United States (US) EDs which are largely accurate in identifying acute abdominal disease processes, including laboratory tests (liver function, lactic acid), hepatobiliary ultrasound, and multiple modalities of computed tomography (CT) scan (noncontrast, contrast, angiography, and enterography) [22, 23].

This high morbidity and mortality among older adults with abdominal pain, as well as the uncertain but decreased accuracy of history and physical exams, has translated into a high index of suspicion for pathology among emergency physicians, with a low threshold for CT imaging and hospitalization. 1 in 4 patients 65–74 years old and 1 in 3 patients 75 years and older were admitted in 2021 (versus 13% for all patients in the ED) [1]. Nationally, a large and growing proportion of ED patients with abdominal pain received CT imaging: 42.6% of all aged patients, and over 2 in 3 older adults (≥ 65 years) in 2019 [2].

These crucial differences between the pathophysiology, signs, symptoms, and epidemiology of abdominal pain presentations between younger and older adults raise the possibility that, despite the perennial increase in CT utilization in the ED raising concern for overutilization in the overall population of ED visits [24, 25], emergency clinicians may not have sufficiently increased their index of suspicion among older patients in the ED with abdominal pain. For example, older patients with abdominal pain are nearly half as likely to be triaged to the “emergent” (ESI2) category compared to older adults with other chief complaints in the ED, but have similar hospitalization, ICU, and mortality rates, and far higher rates of immediate surgical intervention [3]. A prospective cohort of French ED patients aged 75+ revealed that obtaining a CT on every patient with abdominal pain resulted in diagnoses and hospitalizations for disease that was not clinically suspected, but did not examine patients less than 75 years of age [26].

In this study, we aimed to assess to what extent emergency clinicians have accounted for the increased risks and uncertainty of abdominal pain in older patients relative to younger patients through their index of suspicion and CT imaging. To test these hypotheses, we conducted a secondary, retrospective analysis of a prospective cohort of patients presenting with non‐traumatic abdominal pain to an ED at a US community hospital over a 10‐month period.

## 2 Methods

This study uses data collected by the authors (AM and GK) from a previously published prospective cohort study of 1171 emergency department visits with a chief complaint of abdominal pain that was conducted at a community teaching hospital in southwest Baltimore from March 2016 to January 2017 [27]. The present analysis was approved by the institutional review board of the University of Pennsylvania. Informed consent was waived by the institutional review board.

The study included all patient visits related to non‐traumatic abdominal pain within the 11‐month period, excluding individuals whose age was under 18 years old; 2 patients under the age of 18 were excluded. Patients were categorized into three age groups prior to analysis: 18–39 years, 40–59 years, and 60 years and older. This age stratification was adapted from prior studies of this cohort for consistency and aimed to accommodate the limited number of older adults in the data set with adequate power in each group.

Data extracted by the original study authors from the electronic health record (EHR) included demographic information (age, race, gender, height, weight, and BMI), laboratory results (white blood count, hemoglobin, hematocrit, bicarbonate), medical history (prior abdominal surgeries and presence of diabetes mellitus), and imaging ordered (CT, MRI, X‐ray). The original study was designed and conducted by general surgeon‐researchers (GK and AM) who selected comorbidities based on surgeons' concern for increased risk of operative conditions [27]. Additional variables were obtained by surveying emergency clinicians via a structured form for each study participant in advance of obtaining CT scan results, including reported symptoms during visits (abdominal pain region, nausea, diarrhea, constipation), exam findings (tenderness, rebound tenderness), and an “index of suspicion” collected for any acute abdominal pathology (i.e., clinician gestalt) on a scale of 1–10, with 10 representing the strongest suspicion for an acute abdominal pathology, which we categorize as high, medium, or low [27].

Final CT reports were subsequently evaluated by the original study authors to categorize findings as either acute or non‐acute on the basis of clinical judgment by a surgeon researcher. Renal or ureteral stones with any degree of hydronephrosis were classified in the original study as acute. For this study because these are typically mild and self‐resolving, to increase interpretability and comparability across age groups, we only considered renal calculi with evidence of infection (e.g., noted to be infected, simultaneous diagnosis of UTI or pyelonephritis, or temperature ≥ 38°C) to be acute and actionable, and all other renal calculi to be non‐acute, as they do not require specific management outside of symptom control; uninfected ureteral stones requiring intervention (e.g., stenting or lithotripsy) would be captured as surgery. Additional outcomes recorded were instances of surgery and hospital admission.

Emergency General Surgery (EGS) conditions were identified using the American Association for the Surgery of Trauma (AAST) consensus diagnosis list and mapped to ICD‐10‐CM using the CMS/CDC General Equivalence Mappings (GEMs) [28]. Non‐specific EGS codes were excluded, including abdominal pain, and renal colic.

A composite adverse outcome variable was created to indicate any acute actionable CT findings, an Emergency General Surgical diagnosis, hospital admission, or surgery performed.

To describe the concentration of diagnoses among our patient cohort, the Herfindahl–Hirschman Index (HHI) was calculated by squaring the percentage share of each diagnosis among the total for that age group and then summing these squared values. A high HHI indicates a high concentration of a single diagnosis, whereas an HHI close to zero suggests a more diverse range of diagnoses [29].

Adjustment was performed via linear probability model regression. To test for the proportion of variation explained by additional covariates, we calculated the *R* ^2^ from the progressive addition of sets of covariates to a series of linear probability model regressions.

Analysis was conducted in R 4.4.0, with 95% confidence intervals (CI) for likelihood ratios [30]. Given the number of comparisons made in the study, two‐tailed *p* values from unadjusted analyses are reported but not interpreted for statistical significance.

## 3 Results

We analyzed 1169 patient visits to a community teaching hospital in southwest Baltimore. Of these, 229 (19.6%) were aged 60 years or older. Full demographics by age subgroup are reported in Table 1.

**TABLE 1:** Selected cohort characteristics: Demographics and history.

	18–39 years (*N* = 547)	40–59 years (*N* = 393)	≥ 60 years (*N* = 229)	*p*	Overall (*N* = 1169)
**Demographic**					
Age				< 0.001	
Median	28	50	69		41
Male				< 0.001	
	160 (29.3%)	171 (43.5%)	97 (42.4%)		428 (36.6%)
Race				< 0.001	
White	161 (29.4%)	166 (42.2%)	118 (51.5%)		445 (38.1%)
Black	328 (60.0%)	196 (49.9%)	101 (44.1%)		625 (53.5%)
Hispanic	32 (5.9%)	18 (4.6%)	4 (1.7%)		54 (4.6%)
Asian	24 (4.4%)	13 (3.3%)	3 (1.3%)		40 (3.4%)
Other	2 (0.4%)	0 (0.0%)	3 (1.3%)		5 (0.4%)
BMI				< 0.001	
Underweight	11 (2.0%)	6 (1.5%)	3 (1.3%)		20 (1.7%)
Normal	158 (28.9%)	75 (19.1%)	36 (15.7%)		269 (23.0%)
Overweight	92 (16.8%)	88 (22.4%)	78 (34.1%)		258 (22.1%)
Obese	84 (15.4%)	81 (20.6%)	44 (19.2%)		209 (17.9%)
Morbidly obese	113 (20.7%)	84 (21.4%)	23 (10.0%)		220 (18.8%)
Not obtained	89 (16.3%)	59 (15.0%)	45 (19.7%)		193 (16.5%)
**Past Medical/Surgical History**					
History of diabetes mellitus				< 0.001	
	29 (5.3%)	75 (19.1%)	76 (33.2%)		180 (15.4%)
History of prior abdominal surgery				< 0.001	
	338 (61.8%)	279 (71.0%)	178 (77.7%)		795 (68.0%)

Clinical characteristics for the cohort of visits are reported in Table 2. Notable elements of the history and physical exam differed by age, including more diffuse pain (22.6%, 28.1%, 30.2% of patients aged 18–39, 40–59, and ≥ 60 respectively) and epigastric pain (10.3%, 12.2%, 13.3%) in older patients, similar right upper quadrant (5.0%, 4.1%, 4.9%) but less right lower quadrant (8.3%, 9.4%, 3.6%) and more left lower quadrant pain (5.5%, 7.7%, and 8.9%; *p* < 0.001 across all pain location comparisons). Older patients also reported a trend toward less nausea or vomiting (57.4%, 59.8%, and 50.2%; *p* = 0.078), and less diarrhea (19.7%, 22.6%, 15.3%; *p* = 0.082). Constipation was highest among the oldest patients (5.9%, 7.4%, 12.2%; *p* = 0.017). Older patients were more likely to have a history of diabetes (5.3%, 19.1%, and 33.2%; *p* < 0.001), or prior abdominal surgery (61.8%, 71.0%, and 77.7%; *p* < 0.001). Fewer older adults had abdominal tenderness on physical exam (61.8%, 66.2%, and 53.7%; *p* = 0.009).

**TABLE 2:** Summary statistics of selected cohort characteristics, by age group.

	18–39 years (*N* = 547)	40–59 years (*N* = 393)	≥ 60 years (*N* = 229)	*p*	Overall (*N* = 1169)
**Clinical signs, symptoms, and impression**					
Region of abdomen				< 0.001	
Diffuse	123 (22.6%)	110 (28.1%)	68 (30.2%)		301 (25.9%)
Multiple focal	106 (19.5%)	66 (16.8%)	39 (17.3%)		211 (18.2%)
Right upper quadrant	27 (5.0%)	16 (4.1%)	11 (4.9%)		54 (4.7%)
Right lower quadrant	45 (8.3%)	37 (9.4%)	8 (3.6%)		90 (7.8%)
Left upper quadrant	9 (1.7%)	4 (1.0%)	6 (2.7%)		19 (1.6%)
Left lower quadrant	30 (5.5%)	30 (7.7%)	20 (8.9%)		80 (6.9%)
Right flank	37 (6.8%)	26 (6.6%)	9 (4.0%)		72 (6.2%)
Left flank	19 (3.5%)	23 (5.9%)	16 (7.1%)		58 (5.0%)
Periumbilical	27 (5.0%)	16 (4.1%)	4 (1.8%)		47 (4.0%)
Epigastric	56 (10.3%)	48 (12.2%)	30 (13.3%)		134 (11.5%)
Suprapubic	65 (11.9%)	16 (4.1%)	14 (6.2%)		95 (8.2%)
Nausea/Vomiting				0.078	
	314 (57.4%)	235 (59.8%)	115 (50.2%)		664 (56.8%)
Diarrhea				0.082	
	108 (19.7%)	89 (22.6%)	35 (15.3%)		232 (19.8%)
Constipation				0.017	
	32 (5.9%)	29 (7.4%)	28 (12.2%)		89 (7.6%)
Tenderness				0.009	
	338 (61.8%)	260 (66.2%)	123 (53.7%)		721 (61.7%)
Rebound tenderness				0.185	
	17 (3.1%)	19 (4.8%)	13 (5.7%)		49 (4.2%)
Index of suspicion				0.085	
Low (1–3)	219 (40.0%)	126 (32.1%)	86 (37.6%)		431 (36.9%)
Medium (4–7)	226 (41.3%)	180 (45.8%)	91 (39.7%)		497 (42.5%)
High (8–10)	97 (17.7%)	81 (20.6%)	45 (19.7%)		223 (19.1%)
Not obtained	5 (0.9%)	6 (1.5%)	7 (3.1%)		18 (1.5%)
**Testing and Labs**					
WBC				0.100	
< 4	13 (2.4%)	12 (3.1%)	8 (3.5%)		33 (2.8%)
4–11	402 (73.5%)	264 (67.2%)	170 (74.2%)		836 (71.5%)
> 11	104 (19.0%)	103 (26.2%)	41 (17.9%)		248 (21.2%)
Not obtained	28 (5.1%)	14 (3.6%)	10 (4.4%)		52 (4.4%)
Hemoglobin				0.040	
< 7	1 (0.2%)	0 (0.0%)	4 (1.7%)		5 (0.4%)
≥ 7	519 (94.9%)	379 (96.4%)	215 (93.9%)		1113 (95.2%)
Not obtained	27 (4.9%)	14 (3.6%)	10 (4.4%)		51 (4.4%)
Bicarbonate				0.430	
< 22	60 (11.0%)	54 (13.7%)	20 (8.7%)		134 (11.5%)
22–32	449 (82.1%)	318 (80.9%)	193 (84.3%)		960 (82.1%)
> 32	1 (0.2%)	1 (0.3%)	1 (0.4%)		3 (0.3%)
Not obtained	37 (6.8%)	20 (5.1%)	15 (6.6%)		72 (6.2%)
CT scan performed				< 0.001	
	228 (41.7%)	260 (66.2%)	162 (70.7%)		650 (55.6%)
**Outcome and disposition**					
Composite adverse outcome				< 0.001	
	149 (27.2%)	193 (49.1%)	132 (57.6%)		474 (40.5%)
Acute findings on CT				< 0.001	
	42 (18.4%)	81 (31.2%)	61 (37.7%)		184 (28.3%)
Surgery performed				0.002	
	25 (4.6%)	35 (9.0%)	24 (10.6%)		84 (7.2%)
Any EGS diagnosis				< 0.001	
	104 (19.0%)	119 (30.3%)	80 (34.9%)		303 (25.9%)
Admitted				< 0.001	
	66 (12.1%)	110 (28.0%)	86 (37.6%)		262 (22.4%)

Emergency clinicians reported similar indices of suspicion across the age subgroups, with 19.7% of patients ≥ 60 years old rated as high (vs 19.1% overall; *p* = 0.085; Table 2 and Figure S1). However, CT ordering increased substantially by age group, with 41.7%, 66.2%, and 70.7% of patients aged 18–39, 40–59, and ≥ 60 receiving CT imaging (*p* < 0.001). For every 3 patients with tenderness there were approximately 2 CT scans ordered among 18–39 year olds, 3 CTs among 40–59, and 4 CTs among ≥ 60 year old patients.

Despite increased CT imaging for older adults, the yield/test positivity of CT imaging also increased, with 18.4%, 31.2%, and 37.7% of CT scans showing acute findings in patients aged 18–39, 40–59, and ≥ 60, respectively (*p* < 0.001; Table 2). Similarly, acute surgical intervention increased by age group (4.6%, 9.0%, and 10.6%; *p* = 0.002) and higher proportions were admitted in the older age groups (12.1%, 28.0%, and 37.6%; *p* < 0.001). When these four outcomes were combined, the composite adverse outcome increased with age as well (27.2%, 49.1%, and 57.6%; *p* < 0.001).

Using the composite adverse outcome of acute findings on CT, surgery performed, EGS diagnosis, or admission to the hospital (Table 3), a physical exam finding of tenderness was less sensitive for adverse outcomes in older adults [sensitivity 0.73 (95% CI: 0.66–0.80), 0.73 (0.66–0.79), and 0.58 (0.49–0.66) by age group respectively]. A finding of rebound tenderness was uncommon but, when present, remained highly specific across age groups [specificity 0.98 (95% CI: 0.96–0.99), 0.96 (0.92–0.98), and 0.98 (0.93–0.99)]. Table S1 reports the components of the composite adverse outcome separately.

**TABLE 3:** Unadjusted diagnostic test characteristics of selected history and physical examination findings using adverse outcome composite as criterion standard, by age group.

Variable	Age	Total *N*	Sensitivity (95% CI)	Specificity (95% CI)
Diffuse abdominal pain	[18–39]	544	0.26 (0.19–0.33)	0.79 (0.74–0.82)
Diffuse abdominal pain	[40–59]	392	0.31 (0.25–0.38)	0.75 (0.69–0.81)
Diffuse abdominal pain	[≥ 60]	225	0.30 (0.22–0.38)	0.69 (0.59–0.77)
Tenderness	[18–39]	547	0.73 (0.66–0.80)	0.43 (0.38–0.47)
Tenderness	[40–59]	393	0.73 (0.66–0.79)	0.41 (0.34–0.47)
Tenderness	[≥ 60]	229	0.58 (0.49–0.66)	0.52 (0.42–0.61)
Rebound tenderness	[18–39]	547	0.05 (0.03–0.10)	0.98 (0.96–0.99)
Rebound tenderness	[40–59]	393	0.05 (0.03–0.09)	0.96 (0.92–0.98)
Rebound tenderness	[≥ 60]	229	0.08 (0.05–0.14)	0.98 (0.93–0.99)
Abnormal white blood count	[18–39]	519	0.31 (0.24–0.39)	0.81 (0.76–0.84)
Abnormal white blood count	[40–59]	379	0.41 (0.34–0.48)	0.80 (0.74–0.85)
Abnormal white blood count	[≥ 60]	219	0.32 (0.24–0.40)	0.91 (0.83–0.95)

*Note:* The idea is that each color represents an age group, to facilitate comparison across ages and across findings.

In adjusted analyses (Table S2), few individual predictors were statistically significant. However, the more information provided to a regression model (History and Vital signs, Exam, Laboratory testing, Index of Suspicion, Imaging Ordered), the higher the proportion of variation in the composite adverse outcome the model was able to explain. The overall *R* ^2^ indicated that including all the demographic and clinical variables explained 22% of the variation in the composite adverse outcome for younger adults, 25% for middle‐aged adults, and 37% for older adults (Table 4). Notably, after adjustment, for older adults a 1 point increase in index of suspicion (out of a possible 10 points) raised the probability of a composite adverse event by 4.2 percentage points (*p* < 0.01, Table S2).

**TABLE 4:** Proportion of the variation in adverse outcome composite explained by serial addition of history and vital signs, physical exam findings, laboratory testing, index of suspicion, and whether imaging was ordered, by age group.

	Adjusted *R* ^2^		
	18–39 years	40–59 years	≥ 60 years
History and vitals	5.0%	7.5%	11.9%
+ Physical Exam	10.1%	10.4%	15.4%
+ Lab testing	16.8%	21.7%	28.2%
+ Index of suspicion	21.0%	23.6%	32.7%
+ Imaging ordered	22.3%	24.7%	36.6%

*Note:* Each cell presents the *R* ^2^ value from a separate linear probability regression model, multiplied by 100 for ease of interpretation as a percentage. Each row added an additional group of variables to a regression model and includes all groups of variables above it. For instance, the third row “+ Lab Testing” denotes a model with variables for: history and vitals, exam findings, and lab testing. Variables in the group “history and vitals” include: diabetes diagnosis, prior abdominal surgery, BMI group, nausea and/or vomiting, diarrhea, or fever. Variables in the group “Exam” include: abdominal pain location, any tenderness, and rebound tenderness. Variables in the group “Lab testing” include: hemoglobin group, white blood cell group, and bicarbonate group. The group “Index of suspicion” includes only the index of suspicion variable, modeled linearly. Variables in the group “Imaging ordered” include: Ultrasound and MRI; CT ordering was excluded because its outcome was included in the composite adverse outcome. Full regression results are available in Table S2.

**FIGURE 1:**
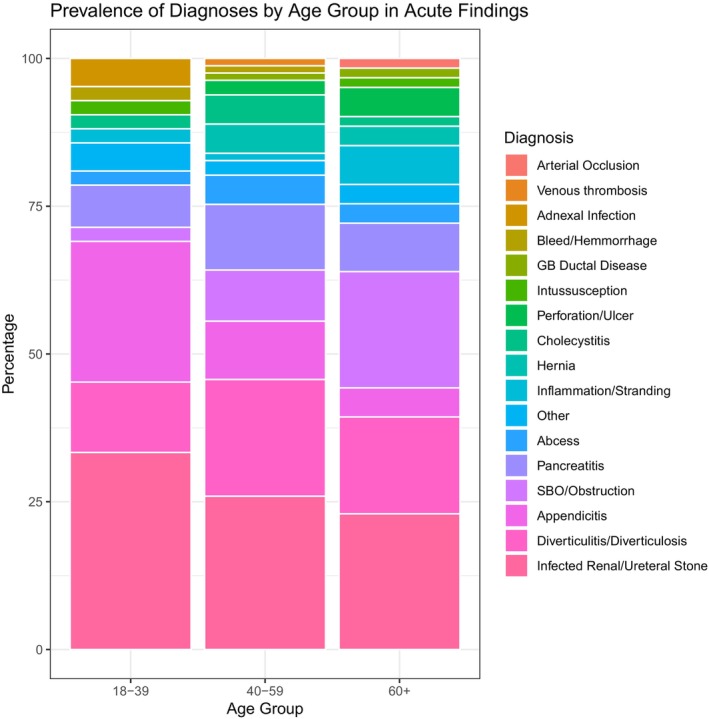
Prevalence of diagnoses by age group, among visits resulting in acute findings.

The incidence of acute actionable CT findings by age group is shown in Figure 1. Acute findings were more concentrated in fewer diagnoses in younger adults. For 18–39 year olds, only 3 diagnoses account for 69.0% of CT findings (infected ureteral stone, acute diverticular disease, and appendicitis). The top 3 diagnoses accounted for 56.8% of findings in 40–59 year olds and 59% in ≥ 60 year olds. The HHI trended toward a decrease with age (1950, 1448, and 1384). There was 1 patient (age 60+) diagnosed with arterial occlusion, and no patients with a specific diagnosis of mesenteric ischemia.

## 4 Discussion

In this retrospective analysis of 1169 patient visits to a community teaching hospital in southwest Baltimore who presented to the ED with a chief complaint of abdominal pain, we found that compared to young adults older adults presented with more nonspecific signs and symptoms. Clinicians reported a similar index of suspicion across age categories—and patients had less nausea/vomiting and tenderness at older ages—but clinicians ordered testing substantially more for older adults. Despite this increased rate of testing, the test positivity—defined alternatively as acute findings on CT, admission, or operative management—was higher for older adults.

Our findings contribute to the body of literature and geriatricians' clinical experience regarding core geriatric hypotheses that older adults, especially those with abdominal pain, present diagnostic challenges—limited history and physical exam, more potential acute diagnoses, and high morbidity and mortality. While contemporary ED management suggests we have made progress, it is unclear if it is sufficient.

Interestingly, we see a discrepancy between clinicians' report of their index of suspicion and testing decisions. Despite reporting nearly identical indices of suspicion by age, emergency clinicians in our study were substantially more likely to order CT imaging for older patients. Clinicians may be normalizing risk by age when they report their index of suspicion.

Even with this increase, however, the yield of CT imaging—the proportion of CTs with acute, actionable findings—also increased substantially across age groups. Despite a 70% relative increase in scan rate for the oldest vs. youngest adults, the yield still more than doubled. While the optimal test positivity that would balance the risks of testing, overdiagnosis, and resource use is unknown, it is unlikely that older adult test positivity being nearly double the test positivity for younger adults is optimal. While this may suggest that the imaging threshold is too low for younger patients, in this study, 41.7% of patients 18–39 had CT imaging with a yield of 18.4%, which does not strike us as inappropriately low. Although there are increases in CT scanning in the past decades [2, 24], which has raised concern for overutilization [24], our results suggest the distribution and appropriateness of CT imaging almost certainly varies by age group.

When comparing 40–59 year olds to older adults, CT ordering only increased from 66.2% to 70.7% (6.8% relative increase), with the CT yield increasing from 31.2% to 37.7% (a 21% relative increase) and a similar (9% vs. 10.6%; 17.8% relative increase) in surgical intervention. This, combined with other research teams' findings in 75+ year olds [26], suggests that further increasing CT imaging for older patients with abdominal pain may be warranted to identify nonoperative processes which may benefit from specific therapies (e.g., antibiotics). The difference in CT ordering in the middle and older age groups in our study is fairly small; this may be at least partially related to the age cutoffs used in this study, defining older adults at the relatively young age of 60 for consistency with previous studies in this cohort and due to there being relatively few older adults in this dataset, limiting power.

### 4.1 History and Physical Examination Accuracy

Our findings bring additional evidence to the geriatric hypothesis that history and physical exam (H&P) are less accurate in older patients, with fewer older patients reporting focal abdominal pain (with the exception of left lower quadrant pain, possibly related at least in part to a higher incidence of diverticulitis among older patients) [31]. Lower rates of nausea/vomiting and diarrhea in older patients did not reach traditional statistical significance thresholds in our cohort. Older patients did report more constipation, consistent with literature showing ED presentations for constipation increase with age and sometimes reflect bowel obstruction or other significant disease [32, 33].

The oldest patients in our cohort had the lowest rates of abdominal tenderness, yet still had CTs ordered at higher rates, consistent with emergency clinicians qualitatively incorporating the core geriatric lessons from prior studies and teachings on the inaccuracy of abdominal exams in older patients.

Our results add to the body of literature on limitations of history and physical in older adults with specific abdominal presentations dating back decades for older adults with cholecystitis and appendicitis [34, 35]. Across all conditions, Laurell and colleagues found that rebound tenderness and rigidity were less common in older adults and that older adults were more likely to have their abdominal pain misdiagnosed in the ED and at hospital discharge [36]. Given these nonspecific signs and symptoms, previous studies demonstrate that CT has significant utility in older adults [26, 37]. In light of these missed findings, the high test positivity seen on CT of older adults in the present cohort suggests that additional disease would be diagnosed were cross‐sectional imaging selectively targeted to older adults with abdominal pain.

### 4.2 Increased Diagnoses List

Older adults experienced a greater diversity of diagnoses as measured by HHI, consistent with their hypothesized greater diagnostic complexity. If there were only five diagnoses, each with 20% of the patient visits, the HHI would be 2000, similar to that observed for younger adults. The observed HHI difference between older and younger adults is approximately equivalent to redistributing the cases from 2 of those 5 diagnoses instead among 10 alternative diagnoses (i.e., the top 3 diagnoses each with 20% of patients, and the next 10 diagnoses with 2% each)—a far longer tail of clinical complexity for the clinician to consider.

### 4.3 Is Middle Age Closer to Geriatric? Where Is the Cutoff?

Interestingly, our findings suggest that middle age (40–59 year old) patients with abdominal pain are more similar in many respects to patients ≥ 60 years old than to young adults with respect to the incidence of signs and symptoms (excepting nausea/vomiting and abdominal tenderness) and the yield and distribution of CT findings. The prespecified age groups may have contributed to this. This study is limited by relatively few patients ≥ 60. A larger study would allow analyses of the oldest old (85+) and allow viewing age as a continuous risk factor. In addition, patients' goals of care likely differ systematically by age, and there may be selection in older adults with frailty or cognitive impairment in the choice to present to the ED or to undergo imaging.

Nationally, 42.6% of ED visits in 2019 received a CT, compared to 55.6% in our sample in 2016–17 [2]. Visits where CTs were obtained resulted in an EGS diagnosis 17.8% of the time nationally in 2017–19 (compared to our test positivity of 28.3%). Although population differences, age group cutoffs, and definitional differences limit comparability, we see similar patterns of increasing CT imaging and test positivity with age, but with a greater difference between younger and older adults in the present study.

Taken as a whole, these findings confirm classic geriatric hypotheses and argue for a shift in how we approach abdominal pain in older patients, who have lower reserve and are more vulnerable to acute insult due to frailty, multimorbidity, and cognitive impairment.

The differences in presentation, diagnoses, and outcomes for abdominal pain in older adults are so distinct that we propose viewing 'geriatric abdominal pain' as a distinct entity, a 'geriatric chief complaint' which requires distinct differential diagnoses, cognitive processes, and testing pathways. This resembles certain pediatric chief complaints. For instance, an ED presentation of a 28‐year‐old with back pain is likely benign if red flags are not present, whereas a presentation for pediatric back pain is higher risk and has a differential that includes malignancy and infection [38].

This conceptualization can help primary care and emergency clinicians “right‐size” the workup for older patients with abdominal pain through a higher index of suspicion and lower threshold for CT imaging (i.e., accounting for history and physical exam limitations and higher morbidity and mortality); consideration of alternate diagnostic modalities (e.g., CT angiography, if mesenteric ischemia is suspected [39]); and, a hospitalization decision which balances potential benefits of observation in older patients with concerning symptoms but unrevealing diagnostic testing against iatrogenic harm such as delirium. Relatedly, while we do not have specific data on iatrogenic harm from CT imaging in our participants, expanding CT imaging (especially for younger patients) increases risks such as radiation exposure and need for follow up and related patient stress regarding incidental findings, and increases ED capacity strain. Other symptom‐based chief complaints will similarly fit into the geriatric chief complaint model; potential candidates include dizziness and shortness of breath.

## 5 Limitations

Our study has a number of limitations. This data is from a single, urban, community hospital, which may not be nationally representative due to differences in local population and in clinical practice (e.g., CT ordering, disposition, outpatient referral patterns and follow‐up availability). Compared to national ED data, this population of ED visitors was younger but more diverse along racial and ethnic dimensions. The data lacked adequate power to investigate differences in age groups among adults ≥ 60. The data is from a single 10‐month period prior to the COVID‐19 pandemic. While we do not mean to downplay or diminish the pain and importance of renal stones—particularly from the patients' perspective—we only considered renal calculi with evidence of infection to be an adverse outcome, as uncomplicated stones, which can be remarkably painful, are not inherently dangerous per se. Further, uninfected ureteral stones requiring admission for pain control or intervention (e.g., stenting or lithotripsy) are captured under admission and/or surgery, patient‐oriented outcomes. The cohort as abstracted from the EHR in the initial study did not include lactic acid, a potentially important marker of bowel ischemia and sepsis, nor hepatobiliary or pancreatic labs. Further, cohort data did not differentiate between CT modalities such as non‐contrast, contrast, and angiography. Therefore we cannot determine whether the low rate of diagnoses consistent with mesenteric ischemia were due to a lack of definitive testing or the diagnosis being rare. Finally, due to data limitations we were unable to assess outcomes extending beyond the index admission (e.g., ED revisits, 30‐day mortality, or missed diagnoses among patients with negative CTs); instead we use CT test positivity among those imaged and a composite outcome of adverse events, which enables comparisons across age groups but cannot determine an ideal test rate.

## 6 Conclusions

In this study, we found that while emergency clinicians account qualitatively for the increased risks and uncertainty of abdominal pain in older patients by increasing CT imaging, the yield of CT imaging is substantially higher in older adults. Additional testing would likely identify acute, intervenable findings. This diagnostic imbalance between age groups may represent an under‐appreciation of the risks of older adults, an under‐valuation of identification of acute pathology in older adults, or an over‐reliance on history and physical examination findings. Informative signs and symptoms are less common in older adults despite higher rates of acute pathology, consistent with the geriatric hypothesis that older adults present with less specific symptoms.

## Conflicts of Interest

Ari B. Friedman reports funding from the National Institute on Aging (1‐K23‐AG080061), which supported this work, and unrelated funding from the Emergency Medicine Foundation. MNA reports no conflicts of interest. Liliana A. Schadt reports no conflicts of interest. Yang Li reports no conflicts of interest. Anne R. Cappola reports funding from the NIA. Rachel R. Kelz reports funding from the NIA. Ula Y. Hwang reports conflicts of interest as one of the editors of the Special Issue submission (recused), and reports funding from the John A. Hartford Foundation, the West Health Institute, and the NIA. Gopal Kowdley reports no conflicts of interest. Aubrey Mwinyogle reports no conflicts of interest. N. Seth Trueger receives support from the American Medical Association as Digital Media Editor for JAMA Network Open.

## Supporting information


**Figure S1:** Index of suspicion compared to CT test ordering and acute findings on CT.
**Table S1:** Test characteristics of selected history, examination, and testing using EGS diagnosis, acute finding on CT, surgery, and admission as criterion standards.
**Table S2:** Adjusted analyses: Relationship of variables to composite adverse outcome via linear probability models.
**Table S3:** ICD‐10 codes used to define Emergency General Surgery diagnoses, grouped.

## Data Availability

The data that support the findings of this study are available from the the original cohort authors upon reasonable request from academic for academic purposes.
