# Supplementary material for: Clinical Features, CT Imaging Decisions and Yield by Age in Adults With Abdominal Pain in the Emergency Department
Source: Acad Emerg Med. 2026 May 10;33:e70299. doi: 10.1111/acem.70299 (PMC13158444; doi:10.1111/acem.70299)
Supplement: Supplementary file 1 — Figure S1: Index of suspicion compared to CT test ordering and acute findings on CT. Table S1: Test characteristics of selected history, examination, and testing using EGS diagnosis, acute finding on CT, surgery, and admission as criterion standards. Table S2: Adjusted analyses: Relationship of variables to composite adverse outcome via linear probability models. Table S3: ICD‐10 codes used to define Emergency General Surgery diagnoses, grouped. [file ACEM-33-0-s001.docx]

**Table S1: Test characteristics of selected history, examination, and testing using EGS diagnosis, acute finding on CT, surgery, and admission as criterion standards.**

**Emergency General Surgical Diagnosis**

| Variable | | Age | truePos | totalDzPos | trueNeg | totalDzNeg | Sensitivity | Specificity | Positive LR | Negative LR |
| --- | --- | --- | --- | --- | --- | --- | --- | --- | --- | --- |
| CT performed | [18-39] | | 53 | 104 | 268 | 443 | 0.51 (0.42 - 0.60) | 0.61 (0.56 - 0.65) | 1.29 (1.02 - 1.60) | 0.81 (0.65 - 0.99) |
| CT performed | [40-59] | | 82 | 119 | 96 | 274 | 0.69 (0.60 - 0.77) | 0.35 (0.30 - 0.41) | 1.06 (0.91 - 1.23) | 0.89 (0.63 - 1.20) |
| CT performed | ≥60 | | 61 | 80 | 48 | 149 | 0.76 (0.66 - 0.84) | 0.32 (0.25 - 0.40) | 1.13 (0.95 - 1.32) | 0.74 (0.44 - 1.14) |
| Diffuse abdominal pain | [18-39] | | 29 | 104 | 346 | 440 | 0.28 (0.20 - 0.37) | 0.79 (0.75 - 0.82) | 1.31 (0.88 - 1.83) | 0.92 (0.80 - 1.03) |
| Diffuse abdominal pain | [40-59] | | 40 | 119 | 203 | 273 | 0.34 (0.26 - 0.43) | 0.74 (0.69 - 0.79) | 1.31 (0.93 - 1.80) | 0.89 (0.76 - 1.03) |
| Diffuse abdominal pain | ≥60 | | 24 | 80 | 101 | 145 | 0.30 (0.21 - 0.41) | 0.70 (0.62 - 0.77) | 0.99 (0.63 - 1.49) | 1.01 (0.83 - 1.19) |
| Tenderness | [18-39] | | 74 | 104 | 179 | 443 | 0.71 (0.62 - 0.79) | 0.40 (0.36 - 0.45) | 1.19 (1.02 - 1.37) | 0.71 (0.50 - 0.96) |
| Tenderness | [40-59] | | 88 | 119 | 102 | 274 | 0.74 (0.65 - 0.81) | 0.37 (0.32 - 0.43) | 1.18 (1.02 - 1.35) | 0.70 (0.48 - 0.97) |
| Tenderness | ≥60 | | 49 | 80 | 75 | 149 | 0.61 (0.50 - 0.71) | 0.50 (0.42 - 0.58) | 1.23 (0.96 - 1.56) | 0.77 (0.54 - 1.04) |
| Rebound tenderness | [18-39] | | 5 | 104 | 431 | 443 | 0.05 (0.02 - 0.11) | 0.97 (0.95 - 0.98) | 1.78 (0.40 - 4.74) | 0.98 (0.93 - 1.02) |
| Rebound tenderness | [40-59] | | 8 | 119 | 263 | 274 | 0.07 (0.03 - 0.13) | 0.96 (0.93 - 0.98) | 1.68 (0.57 - 4.22) | 0.97 (0.91 - 1.02) |
| Rebound tenderness | ≥60 | | 9 | 80 | 145 | 149 | 0.11 (0.06 - 0.20) | 0.97 (0.93 - 0.99) | 4.19 (1.38 - Inf) | 0.91 (0.83 - 0.98) |
| Abnormal white blood count | [18-39] | | 32 | 101 | 333 | 418 | 0.32 (0.23 - 0.41) | 0.80 (0.76 - 0.83) | 1.56 (1.07 - 2.17) | 0.86 (0.74 - 0.98) |
| Abnormal white blood count | [40-59] | | 48 | 117 | 195 | 262 | 0.41 (0.33 - 0.50) | 0.74 (0.69 - 0.79) | 1.60 (1.18 - 2.17) | 0.79 (0.67 - 0.93) |
| Abnormal white blood count | ≥60 | | 24 | 78 | 116 | 141 | 0.31 (0.22 - 0.42) | 0.82 (0.75 - 0.88) | 1.74 (1.05 - 2.89) | 0.84 (0.70 - 0.98) |
| Acuteness of CT Diagnosis | [18-39] | | 24 | 53 | 157 | 175 | 0.45 (0.33 - 0.58) | 0.90 (0.84 - 0.93) | 4.40 (2.58 - 7.80) | 0.61 (0.46 - 0.76) |
| Acuteness of CT Diagnosis | [40-59] | | 49 | 82 | 146 | 178 | 0.60 (0.49 - 0.70) | 0.82 (0.76 - 0.87) | 3.32 (2.34 - 4.89) | 0.49 (0.36 - 0.63) |
| Acuteness of CT Diagnosis | ≥60 | | 38 | 61 | 78 | 101 | 0.62 (0.50 - 0.73) | 0.77 (0.68 - 0.84) | 2.74 (1.84 - 4.28) | 0.49 (0.33 - 0.66) |

**Acute finding on CT (exclusive of renal stone)**

| Variable | Age | truePos | totalDzPos | trueNeg | totalDzNeg | Sensitivity | Specificity | Positive LR | Negative LR |
| --- | --- | --- | --- | --- | --- | --- | --- | --- | --- |
| Diffuse abdominal pain | [18-39] | 6 | 42 | 149 | 186 | 0.14 (0.07 - 0.28) | 0.80 (0.74 - 0.85) | 0.72 (0.24 - 1.45) | 1.07 (0.91 - 1.22) |
| Diffuse abdominal pain | [40-59] | 22 | 81 | 137 | 178 | 0.27 (0.19 - 0.38) | 0.77 (0.70 - 0.82) | 1.18 (0.72 - 1.82) | 0.95 (0.80 - 1.10) |
| Diffuse abdominal pain | ≥60 | 15 | 61 | 68 | 101 | 0.25 (0.15 - 0.37) | 0.67 (0.58 - 0.76) | 0.75 (0.41 - 1.23) | 1.12 (0.91 - 1.36) |
| Tenderness | [18-39] | 29 | 42 | 46 | 186 | 0.69 (0.54 - 0.81) | 0.25 (0.19 - 0.31) | 0.92 (0.71 - 1.12) | 1.25 (0.68 - 2.03) |
| Tenderness | [40-59] | 62 | 81 | 50 | 179 | 0.77 (0.66 - 0.84) | 0.28 (0.22 - 0.35) | 1.06 (0.91 - 1.23) | 0.84 (0.49 - 1.28) |
| Tenderness | ≥60 | 33 | 61 | 34 | 101 | 0.54 (0.42 - 0.66) | 0.34 (0.25 - 0.43) | 0.82 (0.60 - 1.05) | 1.36 (0.91 - 2.02) |
| Rebound tenderness | [18-39] | 4 | 42 | 174 | 186 | 0.10 (0.04 - 0.22) | 0.94 (0.89 - 0.96) | 1.48 (0.23 - 3.92) | 0.97 (0.85 - 1.05) |
| Rebound tenderness | [40-59] | 7 | 81 | 170 | 179 | 0.09 (0.04 - 0.17) | 0.95 (0.91 - 0.97) | 1.72 (0.56 - 4.98) | 0.96 (0.88 - 1.03) |
| Rebound tenderness | ≥60 | 4 | 61 | 95 | 101 | 0.07 (0.03 - 0.16) | 0.94 (0.88 - 0.97) | 1.10 (0.26 - 4.96) | 0.99 (0.90 - 1.07) |
| Abnormal white blood count | [18-39] | 15 | 42 | 125 | 181 | 0.36 (0.23 - 0.51) | 0.69 (0.62 - 0.75) | 1.15 (0.68 - 1.77) | 0.93 (0.70 - 1.16) |
| Abnormal white blood count | [40-59] | 43 | 81 | 132 | 175 | 0.53 (0.42 - 0.64) | 0.75 (0.69 - 0.81) | 2.16 (1.57 - 3.10) | 0.62 (0.47 - 0.78) |
| Abnormal white blood count | ≥60 | 21 | 61 | 79 | 99 | 0.34 (0.24 - 0.47) | 0.80 (0.71 - 0.86) | 1.70 (0.99 - 2.95) | 0.82 (0.65 - 1.00) |

**Surgery**

| Variable | Age | truePos | totalDzPos | trueNeg | totalDzNeg | Sensitivity | Specificity | Positive LR | Negative LR |
| --- | --- | --- | --- | --- | --- | --- | --- | --- | --- |
| CT performed | [18-39] | 17 | 25 | 311 | 521 | 0.68 (0.48 - 0.83) | 0.60 (0.55 - 0.64) | 1.69 (1.19 - 2.17) | 0.54 (0.25 - 0.86) |
| CT performed | [40-59] | 24 | 35 | 120 | 355 | 0.69 (0.52 - 0.81) | 0.34 (0.29 - 0.39) | 1.04 (0.78 - 1.27) | 0.93 (0.48 - 1.44) |
| CT performed | ≥60 | 21 | 24 | 64 | 202 | 0.88 (0.69 - 0.96) | 0.32 (0.26 - 0.38) | 1.28 (1.03 - 1.49) | 0.40 (0.01 - 0.91) |
| Diffuse abdominal pain | [18-39] | 5 | 25 | 400 | 518 | 0.20 (0.09 - 0.39) | 0.77 (0.73 - 0.81) | 0.88 (0.27 - 1.69) | 1.04 (0.80 - 1.23) |
| Diffuse abdominal pain | [40-59] | 7 | 35 | 254 | 354 | 0.20 (0.10 - 0.36) | 0.72 (0.67 - 0.76) | 0.71 (0.28 - 1.27) | 1.12 (0.90 - 1.30) |
| Diffuse abdominal pain | ≥60 | 8 | 24 | 139 | 198 | 0.33 (0.18 - 0.53) | 0.70 (0.64 - 0.76) | 1.12 (0.51 - 1.89) | 0.95 (0.65 - 1.22) |
| Tenderness | [18-39] | 18 | 25 | 201 | 521 | 0.72 (0.52 - 0.86) | 0.39 (0.34 - 0.43) | 1.17 (0.85 - 1.45) | 0.73 (0.30 - 1.23) |
| Tenderness | [40-59] | 29 | 35 | 127 | 355 | 0.83 (0.67 - 0.92) | 0.36 (0.31 - 0.41) | 1.29 (1.06 - 1.50) | 0.48 (0.16 - 0.88) |
| Tenderness | ≥60 | 14 | 24 | 95 | 202 | 0.58 (0.39 - 0.76) | 0.47 (0.40 - 0.54) | 1.10 (0.71 - 1.51) | 0.89 (0.47 - 1.36) |
| Rebound tenderness | [18-39] | 2 | 25 | 506 | 521 | 0.08 (0.02 - 0.25) | 0.97 (0.95 - 0.98) | 2.78 (0.00 - 8.82) | 0.95 (0.81 - 1.03) |
| Rebound tenderness | [40-59] | 1 | 35 | 337 | 355 | 0.03 (0.01 - 0.15) | 0.95 (0.92 - 0.97) | 0.56 (0.00 - 2.30) | 1.02 (0.94 - 1.07) |
| Rebound tenderness | ≥60 | 3 | 24 | 192 | 202 | 0.12 (0.04 - 0.31) | 0.95 (0.91 - 0.97) | 2.52 (0.31 - 8.45) | 0.92 (0.74 - 1.04) |
| Abnormal white blood count | [18-39] | 9 | 25 | 386 | 493 | 0.36 (0.20 - 0.56) | 0.78 (0.74 - 0.82) | 1.66 (0.81 - 2.65) | 0.82 (0.56 - 1.05) |
| Abnormal white blood count | [40-59] | 19 | 35 | 246 | 341 | 0.54 (0.38 - 0.70) | 0.72 (0.67 - 0.77) | 1.95 (1.31 - 2.70) | 0.63 (0.40 - 0.87) |
| Abnormal white blood count | ≥60 | 11 | 24 | 154 | 192 | 0.46 (0.28 - 0.65) | 0.80 (0.74 - 0.85) | 2.32 (1.24 - 3.76) | 0.68 (0.41 - 0.92) |
| Acuteness of CT Diagnosis | [18-39] | 15 | 17 | 184 | 210 | 0.88 (0.66 - 0.97) | 0.88 (0.82 - 0.91) | 7.13 (4.78 - 10.93) | 0.13 (0.00 - 0.35) |
| Acuteness of CT Diagnosis | [40-59] | 20 | 24 | 175 | 235 | 0.83 (0.64 - 0.93) | 0.74 (0.69 - 0.80) | 3.26 (2.38 - 4.26) | 0.22 (0.06 - 0.46) |
| Acuteness of CT Diagnosis | ≥60 | 19 | 21 | 96 | 138 | 0.91 (0.71 - 0.97) | 0.70 (0.61 - 0.77) | 2.97 (2.21 - 4.00) | 0.14 (0.00 - 0.36) |

**Admission**

| Variable | Age | truePos | totalDzPos | trueNeg | totalDzNeg | Sensitivity | Specificity | Positive LR | Negative LR |
| --- | --- | --- | --- | --- | --- | --- | --- | --- | --- |
| CT performed | [18-39] | 41 | 66 | 294 | 481 | 0.62 (0.50 - 0.73) | 0.61 (0.57 - 0.65) | 1.60 (1.26 - 1.97) | 0.62 (0.43 - 0.83) |
| CT performed | [40-59] | 78 | 110 | 101 | 283 | 0.71 (0.62 - 0.79) | 0.36 (0.30 - 0.41) | 1.10 (0.94 - 1.27) | 0.82 (0.57 - 1.12) |
| CT performed | ≥60 | 72 | 86 | 53 | 143 | 0.84 (0.74 - 0.90) | 0.37 (0.30 - 0.45) | 1.33 (1.14 - 1.56) | 0.44 (0.23 - 0.71) |
| Diffuse abdominal pain | [18-39] | 15 | 66 | 370 | 478 | 0.23 (0.14 - 0.34) | 0.77 (0.73 - 0.81) | 1.01 (0.57 - 1.54) | 1.00 (0.85 - 1.13) |
| Diffuse abdominal pain | [40-59] | 39 | 109 | 212 | 283 | 0.36 (0.27 - 0.45) | 0.75 (0.70 - 0.80) | 1.43 (1.01 - 1.95) | 0.86 (0.73 - 0.99) |
| Diffuse abdominal pain | ≥60 | 29 | 86 | 100 | 139 | 0.34 (0.25 - 0.44) | 0.72 (0.64 - 0.79) | 1.20 (0.78 - 1.78) | 0.92 (0.76 - 1.10) |
| Tenderness | [18-39] | 51 | 66 | 194 | 481 | 0.77 (0.66 - 0.86) | 0.40 (0.36 - 0.45) | 1.30 (1.10 - 1.49) | 0.56 (0.32 - 0.84) |
| Tenderness | [40-59] | 83 | 110 | 106 | 283 | 0.76 (0.67 - 0.82) | 0.38 (0.32 - 0.43) | 1.21 (1.04 - 1.38) | 0.66 (0.43 - 0.91) |
| Tenderness | ≥60 | 50 | 86 | 70 | 143 | 0.58 (0.48 - 0.68) | 0.49 (0.41 - 0.57) | 1.14 (0.89 - 1.45) | 0.85 (0.62 - 1.14) |
| Rebound tenderness | [18-39] | 7 | 66 | 471 | 481 | 0.11 (0.05 - 0.20) | 0.98 (0.96 - 0.99) | 5.10 (1.63 - 13.80) | 0.91 (0.82 - 0.98) |
| Rebound tenderness | [40-59] | 6 | 110 | 270 | 283 | 0.06 (0.03 - 0.11) | 0.95 (0.92 - 0.97) | 1.19 (0.34 - 2.98) | 0.99 (0.93 - 1.04) |
| Rebound tenderness | ≥60 | 7 | 86 | 137 | 143 | 0.08 (0.04 - 0.16) | 0.96 (0.91 - 0.98) | 1.94 (0.55 - 6.73) | 0.96 (0.88 - 1.02) |
| Abnormal white blood count | [18-39] | 22 | 65 | 359 | 454 | 0.34 (0.24 - 0.46) | 0.79 (0.75 - 0.83) | 1.62 (1.05 - 2.32) | 0.84 (0.68 - 0.99) |
| Abnormal white blood count | [40-59] | 52 | 110 | 206 | 269 | 0.47 (0.38 - 0.56) | 0.77 (0.71 - 0.81) | 2.02 (1.50 - 2.72) | 0.69 (0.56 - 0.82) |
| Abnormal white blood count | ≥60 | 33 | 86 | 117 | 133 | 0.38 (0.29 - 0.49) | 0.88 (0.81 - 0.93) | 3.19 (1.91 - 5.80) | 0.70 (0.58 - 0.83) |
| Acuteness of CT Diagnosis | [18-39] | 23 | 41 | 168 | 187 | 0.56 (0.41 - 0.70) | 0.90 (0.85 - 0.93) | 5.52 (3.34 - 9.57) | 0.49 (0.32 - 0.66) |
| Acuteness of CT Diagnosis | [40-59] | 43 | 78 | 144 | 182 | 0.55 (0.44 - 0.66) | 0.79 (0.73 - 0.84) | 2.64 (1.87 - 3.81) | 0.57 (0.42 - 0.72) |
| Acuteness of CT Diagnosis | ≥60 | 41 | 72 | 70 | 90 | 0.57 (0.45 - 0.68) | 0.78 (0.68 - 0.85) | 2.56 (1.69 - 4.16) | 0.55 (0.40 - 0.72) |

**Table S2 Adjusted analyses: Relationship of variables to composite adverse outcome via linear probability models.**

Age: [18-39]

| **Dependent variable: adverse outcome** | | | | | | | |
| --- | --- | --- | --- | --- | --- | --- | --- |
|  | History and vitals | | + Exam | | + Laboratory testing | + Index of suspicion | + Ultrasound or MRI ordering |
| dm | 25.3%p** | | 23.4%p* | | 23.9%p* | 19.2%p* | 20.1%p* |
| prior_abd_surg | 9.2%p* | | 7.7%p | | 6.4%p | 6.5%p | 6.3%p |
| bmigroupUnderweight | -11.5%p | | -8.3%p | | -8.4%p | -6.5%p | -6.9%p |
| bmigroupOverweight | -1.3%p | | 0.1%p | | -2.7%p | -2.9%p | -3.1%p |
| bmigroupObese | -7.8%p | | -8.2%p | | -10.0%p | -9.5%p | -10.6%p |
| bmigroupMorbidly obese | -4.2%p | | -4.7%p | | -7.1%p | -6.8%p | -6.5%p |
| nausea_vomit | 10.7%p* | | 7.3%p | | 4.2%p | 4.6%p | 3.6%p |
| diarrhea | -5.5%p | | -6.0%p | | -5.3%p | -4.0%p | -3.4%p |
| temp_grpLow | 10.5%p | | 0.7%p | | 4.6%p | 5.8%p | 6.6%p |
| temp_grpHigh | 19.2%p | | 11.9%p | | 4.1%p | 4.3%p | 3.8%p |
| roap_newEpigastric |  | | 7.9%p | | 6.1%p | 5.2%p | 3.1%p |
| roap_newLeft flank |  | | -2.2%p | | -1.9%p | -2.0%p | -2.0%p |
| roap_newLeft lower quadrant |  | | 10.2%p | | 10.8%p | 11.4%p | 11.6%p |
| roap_newLeft upper quadrant |  | | 5.6%p | | 12.4%p | 12.3%p | 12.1%p |
| roap_newMultiple focal |  | | -2.0%p | | -4.5%p | -6.0%p | -6.9%p |
| roap_newPeriumbilical |  | | -5.9%p | | -4.6%p | -9.4%p | -9.5%p |
| roap_newRight flank |  | | -0.9%p | | 0.1%p | -1.3%p | -1.5%p |
| roap_newRight lower quadrant |  | | 1.4%p | | -1.4%p | -4.9%p | -4.4%p |
| roap_newRight upper quadrant |  | | 12.2%p | | 7.9%p | 5.0%p | 5.9%p |
| roap_newSuprapubic |  | -20.2%p** | | -19.5%p** | | -22.5%p** | -22.1%p** |
| tend |  | 7.5%p | | 6.1%p | | 3.3%p | 2.9%p |
| rbdtend |  | 18.7%p | | 13.9%p | | 9.2%p | 9.8%p |
| Hemoglobin_group<7 |  |  | | 48.4%p | | 43.0%p | 42.7%p |
| hemoglobin_groupNot obtained |  |  | | -20.4%p | | -16.6%p | -17.7%p |
| wbc_group< 4 |  |  | | -10.1%p | | -11.6%p | -11.2%p |
| wbc_group> 11 |  |  | | 15.3%p** | | 13.9%p** | 13.3%p* |
| bicarb_group< 22 |  |  | | 26.0%p** | | 26.5%p** | 27.5%p** |
| bicarb_group> 32 |  |  | | 72.2%p | | 67.4%p | 67.6%p |
| bicarb_groupNot obtained |  |  | | 21.9%p* | | 21.3%p | 21.7%p* |
| idxsus |  | |  | |  | 3.5%p** | 3.2%p** |
| ultrasound |  | |  | |  |  | -0.8%p |
| mri |  | |  | |  |  | 57.2%p** |
| constant | 18.5%p** | | 17.5%p* | | 17.2%p* | 4.0%p | 6.3%p |
|  | | | | | | | |
| AIC | 559 | | 557 | | 536 | 513 | 510 |
| Observations | 452 | | 450 | | 450 | 448 | 448 |
| R2 | 0.050 | | 0.101 | | 0.168 | 0.210 | 0.223 |
| Adjusted R2 | 0.028 | | 0.054 | | 0.111 | 0.153 | 0.164 |
| Residual Std. Error | 0.442 (df = 441) | | 0.437 (df = 427) | | 0.424 (df = 420) | 0.414 (df = 417) | 0.412 (df = 415) |
| F Statistic | 2.315* (df = 10; 441) | | 2.174** (df = 22; 427) | | 2.929** (df = 29; 420) | 3.699** (df = 30; 417) | 3.731** (df = 32; 415) |
| Note: *p<0.05; **p<0.01; ***p<[0.001] | | | | | | | |

Age: [40-59]

| Dependent variable: adverse outcome | | | | | | | |
| --- | --- | --- | --- | --- | --- | --- | --- |
|  | History and vitals | | + Exam | | + Laboratory testing | + Index of suspicion | + Ultrasound or MRI ordering |
| dm | 3.1%p | | 4.3%p | | 5.2%p | 6.3%p | 6.5%p |
| prior_abd_surg | 1.5%p | | 0.3%p | | 4.5%p | 3.2%p | 1.6%p |
| bmigroupUnderweight | 46.7%p* | | 52.1%p* | | 46.1%p* | 40.2%p | 45.8%p* |
| bmigroupOverweight | 3.0%p | | 2.7%p | | -1.8%p | -3.8%p | -2.3%p |
| bmigroupObese | 0.7%p | | 0.6%p | | -8.3%p | -10.6%p | -8.5%p |
| bmigroupMorbidly obese | 4.9%p | | 5.2%p | | -0.7%p | -0.9%p | 0.1%p |
| nausea_vomit | 23.8%p** | | 21.7%p** | | 18.9%p** | 20.0%p** | 19.4%p** |
| diarrhea | -5.3%p | | -3.6%p | | -3.5%p | -2.0%p | -2.3%p |
| temp_grpLow | 14.7%p | | 10.0%p | | 11.5%p | 13.3%p | 14.5%p |
| temp_grpHigh | 9.7%p | | 15.1%p | | 14.2%p | 12.5%p | 13.0%p |
| roap_newEpigastric |  | | -9.7%p | | -7.2%p | -7.5%p | -8.6%p |
| roap_newLeft flank |  | | 3.3%p | | 9.5%p | 10.6%p | 9.0%p |
| roap_newLeft lower quadrant |  | | -13.5%p | | -15.5%p | -16.1%p | -16.3%p |
| roap_newLeft upper quadrant |  | | -36.9%p | | -31.4%p | -30.3%p | -28.0%p |
| roap_newMultiple focal |  | | 2.1%p | | 7.4%p | 7.6%p | 7.5%p |
| roap_newPeriumbilical |  | | -18.7%p | | -16.3%p | -13.7%p | -13.1%p |
| roap_newRight flank |  | | -10.3%p | | -10.5%p | -11.7%p | -12.0%p |
| roap_newRight lower quadrant |  | | 3.5%p | | 2.3%p | 2.0%p | 2.3%p |
| roap_newRight upper quadrant |  | | 13.0%p | | 12.5%p | 9.9%p | 2.3%p |
| roap_newSuprapubic |  | -12.7%p | | -12.8%p | | -12.0%p | -17.9%p |
| tend |  | 12.4%p* | | 6.0%p | | 3.7%p | 3.6%p |
| rbdtend |  | -5.5%p | | -8.9%p | | -9.5%p | -12.6%p |
| hemoglobin_groupNot obtained |  |  | | -59.3%p** | | -56.9%p** | -55.8%p** |
| wbc_group< 4 |  |  | | -1.3%p | | 0.1%p | -0.9%p |
| wbc_group> 11 |  |  | | 30.5%p** | | 29.1%p** | 29.8%p** |
| bicarb_group< 22 |  |  | | -5.2%p | | -7.8%p | -6.9%p |
| bicarb_group> 32 |  |  | | -73.9%p | | -65.1%p | -62.3%p |
| bicarb_groupNot obtained |  |  | | 43.0%p* | | 43.4%p* | 45.1%p** |
| idxsus |  | |  | |  | 1.9%p | 1.8%p |
| ultrasound |  | |  | |  |  | 11.5%p |
| mri |  | |  | |  |  | 15.9%p |
| constant | 33.4%p** | | 21.1%p** | | 29.0%p** | 22.2%p* | 20.6%p |
|  | | | | | | | |
| AIC | 466 | | 1297 | | 447 | 438 | 437 |
| Observations | 322 | | 947 | | 321 | 318 | 318 |
| R2 | 0.075 | | 0.104 | | 0.217 | 0.236 | 0.247 |
| Adjusted R2 | 0.045 | | 0.083 | | 0.142 | 0.159 | 0.165 |
| Residual Std. Error | 0.489 (df = 311) | | 0.474 (df = 924) | | 0.464 (df = 292) | 0.459 (df = 288) | 0.457 (df = 286) |
| F Statistic | 2.529** (df = 10; 311) | | 4.870** (df = 22; 924) | | 2.890** (df = 28; 292) | 3.068** (df = 29; 288) | 3.019** (df = 31; 286) |
| Note: *p<0.05; **p<0.01; ***p<[0.001] | | | | | | | |

Age: ≥60

| Dependent variable: adverse outcome | | | | | | | |
| --- | --- | --- | --- | --- | --- | --- | --- |
|  | History and vitals | | + Exam | | + Laboratory testing | + Index of suspicion | + Ultrasound or MRI ordering |
| dm | 7.9%p | | 10.9%p | | 9.7%p | 15.5%p | 15.9%p* |
| prior_abd_surg | 16.1%p | | 16.2%p | | 13.2%p | 10.5%p | 11.6%p |
| bmigroupUnderweight | 19.8%p | | 23.5%p | | 27.6%p | 30.9%p | 36.2%p |
| bmigroupOverweight | 7.8%p | | 10.6%p | | 11.6%p | 15.3%p | 17.5%p |
| bmigroupObese | 3.2%p | | 1.1%p | | 4.5%p | 2.6%p | 4.0%p |
| bmigroupMorbidly obese | 12.6%p | | 11.7%p | | 15.0%p | 18.4%p | 20.4%p |
| nausea_vomit | 27.7%p** | | 23.5%p** | | 22.4%p** | 20.0%p** | 18.9%p** |
| diarrhea | 1.5%p | | 2.3%p | | 1.7%p | 0.6%p | 2.4%p |
| temp_grpLow | 22.4%p | | 18.3%p | | 21.8%p | 24.3%p | 24.0%p |
| roap_newEpigastric |  | | -7.5%p | | 0.9%p | 0.0%p | -4.9%p |
| roap_newLeft flank |  | | 6.2%p | | 7.9%p | 7.7%p | 8.9%p |
| roap_newLeft lower quadrant |  | | -1.5%p | | 3.8%p | 4.2%p | 5.1%p |
| roap_newLeft upper quadrant |  | | -15.9%p | | -2.7%p | -6.1%p | -5.6%p |
| roap_newMultiple focal |  | | -7.2%p | | -2.1%p | -4.8%p | -9.5%p |
| roap_newPeriumbilical |  | | 11.1%p | | -1.7%p | -5.6%p | -18.3%p |
| roap_newRight flank |  | | -19.9%p | | -12.8%p | -9.2%p | -16.8%p |
| roap_newRight lower quadrant |  | | -10.3%p | | -20.6%p | -25.7%p | -27.2%p |
| roap_newRight upper quadrant |  | | 14.2%p | | 16.5%p | 14.5%p | -2.2%p |
| roap_newSuprapubic |  | 3.4%p | | 8.0%p | | 5.4%p | 4.3%p |
| tend |  | 5.8%p | | 4.5%p | | 0.9%p | 0.9%p |
| rbdtend |  | 27.4%p | | 21.8%p | | 17.2%p | 19.1%p |
| hemoglobin_group<7 |  |  | | 28.9%p | | 13.4%p | 5.7%p |
| hemoglobin_groupNot obtained |  |  | | -56.1%p | | -51.6%p | -44.5%p |
| wbc_group< 4 |  |  | | 5.2%p | | 4.0%p | 3.5%p |
| wbc_group> 11 |  |  | | 33.1%p** | | 31.0%p** | 28.7%p** |
| bicarb_group< 22 |  |  | | 20.4%p | | 17.0%p | 15.5%p |
| bicarb_group> 32 |  |  | | -3.4%p | | -0.7%p | -21.4%p |
| bicarb_groupNot obtained |  | |  | | 41.3%p | 37.4%p | 33.6%p |
| idxsus |  | |  | |  | 4.2%p** | 4.2%p** |
| ultrasound |  | |  | |  |  | 24.0%p* |
| mri |  | |  | |  |  | 28.9%p |
| constant | 26.4%p* | | 25.5%p | | 15.1%p | -0.5%p | -3.7%p |
|  | | | | | | | |
| AIC | 247 | | 260 | | 245 | 232 | 226 |
| Observations | 178 | | 176 | | 176 | 172 | 172 |
| R2 | 0.119 | | 0.154 | | 0.282 | 0.327 | 0.366 |
| Adjusted R2 | 0.072 | | 0.038 | | 0.146 | 0.190 | 0.226 |
| Residual Std. Error | 0.468 (df = 168) | | 0.475 (df = 154) | | 0.447 (df = 147) | 0.436 (df = 142) | 0.427 (df = 140) |
| F Statistic | 2.533** (df = 9; 168) | | 1.333 (df = 21; 154) | | 2.067** (df = 28; 147) | 2.384** (df = 29; 142) | 2.610** (df = 31; 140) |
| Note: *p<0.05; **p<0.01; ***p<[0.001] | | | | | | | |

Diagnostic test characteristics of the fully adjusted model using adverse outcome composite as the criterion standard, by age group.

|  | **Age Group** | | | | |
| --- | --- | --- | --- | --- | --- |
|  | **[18-39]** | | **[40-59]** | | **[60+]** |
| Diabetes Mellitus | 20.0%p* | | 6.5%p | | 15.9%p* |
| Prior abdominal surgery | 6.3%p* | | 1.6%p | | 11.6%p |
| BMI: Underweight | -6.9%p | | 45.8%p* | | 36.2%p |
| BMI: Overweight | -3.1%p | | -2.3%p | | 17.5%p |
| BMI: Obese | -10.6%p | | -8.5%p | | 4.0%p |
| BMI: Morbidly obese | -6.5%p | | 0.1%p | | 20.4%p |
| Nausea/vomiting | 3.6%p | | 19.4%p** | | 18.9%p** |
| Diarrhea | -3.4%p | | -2.3%p | | 2.4%p |
| Low temperature | 6.6%p | | 14.5%p | | 24.0%p |
| High temperature | 3.8%p | | 13.0%p | | N/A |
| Epigastric pain | 3.1%p | | -8.6%p | | -4.9%p |
| Left flank pain | -2.0%p | | 9.0%p | | 8.9%p |
| Left lower quadrant | 11.6%p | | -16.3%p | | 5.1%p |
| Left upper quadrant | 12.1%p | | -28.0%p | | -5.6%p |
| Multiple foci of pain | -6.9%p | | 7.5%p | | -9.5%p |
| Periumbilical | -9.5%p | | -13.1%p | | -18.3%p |
| Right flank | -1.5%p | | -12.0%p | | -16.8%p |
| Right lower quadrant | -4.4%p | | 2.3%p | | -27.2%p |
| Right upper quadrant | 5.9%p | | 2.3%p | | -2.2%p |
| Suprapubic | -22.1%p** | -17.9%p | | 4.3%p | |
| Any tenderness on exam | 2.9%p | 3.6%p | | 0.9%p | |
| Rebound tenderness | 9.8%p | -12.6%p | | 19.1%p | |
| Hemoglobin <7 | 42.7%p | N/A | | 5.7%p | |
| Hemoglobin not obtained | -17.7%p | -55.8%p** | | -44.5%p | |
| WBC < 4 | -11.2%p | -0.9%p | | 3.5%p | |
| WBC > 11 | 13.3%p* | 29.8%p** | | 28.7%p** | |
| Bicarbonate < 22 | 27.5%p** | -6.9%p | | 15.5%p | |
| Bicarbonate > 32 | 67.6%p | -62.3%p | | -21.4%p | |
| Bicarbonate not obtained | 21.7%p* | 45.1%p** | | 33.6%p | |
| Index of suspicion | 3.2%p** | | 1.8%p | | 4.2%p** |
| Ultrasound obtained | -0.8%p | | 11.5%p | | 24.0%p* |
| MRI obtained | 57.2%p** | | 15.9%p | | 28.9%p |
| AIC | 510 | | 437 | | 226 |
| Observations | 448 | | 318 | | 172 |
| R2 | 0.223 | | 0.247 | | 0.366 |
| Adjusted R2 | 0.164 | | 0.165 | | 0.226 |

Notes: Each column presents a separate linear probability regression model. The coefficients give the adjusted association between each variable and the composite adverse outcome of acute actionable CT findings, hospital admission, surgery performed, or an Emergency General Surgical diagnosis. The coefficient of 13.3%p, for instance, means that having leukocytosis, defined by a white cell count above 11, increases the adjusted probability of having one or more of an acute CT finding, hospital admission, surgery, or an EGS diagnosis by 13.3 percentage points. Negative values reduce the probability.”

**Table S3: ICD-10 codes used to define Emergency General Surgery diagnoses, grouped.**

| **EGS Diagnoses (Grouped)** | |
| --- | --- |
| **Diagnostic grouping** | **ICD-10 codes included** |
| **Colorectal / anorectal / stoma-related** | A04.7, A06.2, C18.0–C18.9, C19*, C20*, C7A*, D12.0–D12.9, K50*, K51*, K52.0, K52.1, K52.8, K55.2, K57.2–K57.9, K59.3, K59.4, K60.2–K60.5, K61*, K62.0–K62.9, K63.5, K64.0–K64.9, K94.0–K94.2, L05.0, L05.9, Z43.2–Z43.4 |
| **Peritoneal / intra-abdominal infection or mass** | K65*, K66.1, K68.1, K68.9, R19.0, R19.3, B25.2 |
| **Hepatobiliary / pancreatic** | D37.6, K72.0, K75.0, K75.1, K76.2, K80.0–K80.8, K81.0–K81.9, K82.0–K82.9, K83.0–K83.9, K85.0–K85.9, K86.2, K86.3, K91.5 |
| **Upper gastrointestinal / foregut** | A02.0, C16.0–C16.9, C17.0–C17.3, C17.8–C17.9, D13.1–D13.3, D37.1–D37.3, D37.8–D37.9, D48.3–D48.4, I85.0–I85.1, K22.3, K22.8, K25.0–K25.9, K26.0–K26.9, K27.0–K27.9, K28.0–K28.9, K29.0, K29.2–K29.9, K31.1, K31.5, K31.6–K31.8, R11.1 |
| **Appendiceal / bowel obstruction / other intestinal** | K35.2–K35.8, K36*, K37*, K38.0–K38.9, K56.0–K56.4, K56.6–K56.7, K57.0–K57.1, K63.0–K63.3, K63.8, K92.0–K92.2 |
| **Other abdominal surgical conditions** | N80.5, Q43.0, Z43.1 |

**Figure S1. Index of suspicion compared to CT test ordering and acute findings on CT.**
